# Reactive oxygen species contribute to dysfunction of bone marrow hematopoietic stem cells in aged C57BL/6 J mice

**DOI:** 10.1186/s12929-015-0201-8

**Published:** 2015-10-24

**Authors:** Marcella L. Porto, Bianca P. Rodrigues, Thiago N. Menezes, Sara L. Ceschim, Dulce E. Casarini, Agata L. Gava, Thiago Melo C. Pereira, Elisardo C. Vasquez, Bianca P. Campagnaro, Silvana S. Meyrelles

**Affiliations:** Laboratory of Translational Physiology, Health Sciences Center, Federal University of Espirito Santo, Vitoria, Brazil; Pharmaceutical Sciences Graduate Program, Vila Velha University, Vila Velha, ES Brazil; Nephrology Division, Department of Medicine, Federal University of Sao Paulo, Sao Paulo, SP Brazil; Division of Nephrology, McMaster University, Hamilton, ON Canada; Federal Institute of Education, Science and Technology, Vila Velha, ES Brazil

**Keywords:** Aging, Oxidative stress, Hematopoietic stem cell, DNA damage, Apoptosis, Senescence

## Abstract

**Background:**

Stem cells of intensely regenerative tissues are susceptible to cellular damage. Although the response to this process in hematopoietic stem cells (HSCs) is crucial, the mechanisms by which hematopoietic homeostasis is sustained are not completely understood. Aging increases reactive oxygen species (ROS) levels and inflammation, which contribute to increased proliferation, senescence and/or apoptosis, leading to self-renewal premature exhaustion. In this study, we assessed ROS production, DNA damage, apoptosis, senescence and plasticity in young, middle and aged (2-, 12- and 24-month-old, respectively) C57BL/6 J mice.

**Results:**

Aged HSCs showed an increase in intracellular superoxide anion (1.4-fold), hydrogen peroxide (2-fold), nitric oxide (1.6-fold), peroxynitrite/hidroxil (2.6-fold) compared with young cells. We found that mitochondria and NADPHox were the major sources of ROS production in the three groups studied, whereas CYP450 contributed in middle and aged, and xanthine oxidase only in aged HSCs. In addition, we observed DNA damage and apoptosis in the middle (4.2- and 2-fold, respectively) and aged (6- and 4-fold, respectively) mice; aged mice also exhibited a significantly shorter telomere length (−1.8-fold) and a lower expression of plasticity markers.

**Conclusion:**

These data suggest that aging impairs the functionality of HSCs and that these age-associated alterations may affect the efficacy of aged HSC recovery and transplantation.

## Background

Stem cells are important for the maintenance of functional tissues and organs and have the abilities to self-renew and replace damaged cells [1]. However, this finely tuned regulatory system may become altered with aging [2]. In fact, recent studies have suggested that the ability to successfully regenerate cells/tissues is gradually lost with aging, as justified by multiple physiological changes at the molecular, cellular, organ, and system levels that occur via mechanisms that are not fully understood [3]. Therefore, the mechanisms that lead to biochemical and cellular age-related alterations in the most primitive cells of hematopoietic system are under investigation in some laboratories.

It is well known that hematopoietic stem cells (HSCs) from aged donor mice have negative effects on stem-cell homing and engraftment [4], are more easily mobilized into the peripheral blood [5] and are therapeutically less efficient [6]. Although some researchers have highlighted the impact of aging on HSCs, the molecular events that trigger the biology of aging remain unclear. In both mice and humans, researchers have profiled the expression of genes involved in genomic integrity and transcriptional regulation demonstrating that the direct or epigenetic modulation of these genes (e.g. p53, p66shc, FOXO3, SIRT1, NF-κB) are consequence of high levels of reactive oxygen species (ROS) in different diseases [7, 8]. For example, it is known that high ROS can inhibit SIRT1 and activate p53, contributing to apoptosis and senescence [9–11]. In addition, it was recently demonstrated that high ROS causes mitochondrial disruption, leading to cytochrome c release and the subsequent activation of a protease cascade [12] with consequent impairment of protein repair pathways [13], culminating in loss of cell integrity, DNA fragmentation and premature exhaustion of self-renewal of these cells [14–16].

Interestingly, while ROS at physiological levels is generated mainly by mitochondrial source and secondary by NADPH oxidase (NAPHox) [14, 17, 18], high ROS production can be derived from other additional sources such as cytochrome P450 (CYP450), xanthine oxidase (XO), lipoxygenases and uncoupling nitric oxide synthase [16, 19–21], which when associated to a reduction of antioxidant activities (e.g. SOD, CAT and GPx) [22] leads to a “vicious cycle” of self-inflicted damage. Thus, since oxidative stress might be considered an important cause of bone marrow HSC dysfunction during the aging process [16], the contributions of different pathways in ROS production deserves to be more explored in this HSC source.

In this study, we analyzed the effects of biological aging on the molecular pathways of ROS production in HSCs. Our findings show a novel age-dependent decline in HSC function that correlates with increased ROS production by different sources. These data suggest that aging impairs the functionality and quality of HSCs, affecting the efficacy of aged HSC recovery and its use in transplantation procedures.

## Methods

### Animals

Experiments were performed in young, middle and aged (2-, 12- and 24-month-old, respectively) male C57BL/6 J (C57) mice that were bred and maintained in the Laboratory of Translational Physiology animal facility (Vitoria, ES, Brazil). The mice were fed a standard chow diet and provided water ad libitum. Animals were housed in individual plastic cages with controlled temperature (22 °C) and humidity (60 %) and were exposed to a 12/12 h light–dark cycle. Mice were euthanized with sodium thiopental overdose (100 mg/kg, intraperitoneal injection). Care and use of laboratory animals were in accordance with National Institutes of Health (NIH) guidelines. All experiments were conducted in compliance with the guidelines for biomedical research, as stated by the Brazilian Societies of Experimental Biology, and were approved by the Institutional Ethics Committee - Emescam College of Health Sciences (CEUA-EMESCAM 014/2011).

### Blood and bone marrow samples

The mice were euthanized, blood was collected for a complete blood count (CBC) and the femurs and tibias were removed. The marrow cavities were flushed with sterile Dulbecco’s Modified Eagle Medium (DMEM Sigma-Aldrich, Saint Louis, MO, USA). Cell suspension were placed in culture with DMEM supplemented with 20 % FBS (Gibco Life Technologies, São Paulo, SP, Brazil), 100 U/mL penicillin and 100 μg/mL streptomycin. An aliquot of the cell suspension was counted on a hemocytometer chamber. To enrich the hematopoietic progenitor cell fraction, lineage committed cells were depleted. The cell suspension (1-2×10^7^ cells/mL) was labeled with the Mouse Hematopoietic Stem Cell Enrichment Set containing antibodies against CD3e (CD3 ε chain), CD11b (Integrin αM chain), CD45R/B220, Ly-6G and Ly-6C (Gr-1), and TER-119/Erythroid Cells (Ly-76) (BD Biosciences, San Diego, CA, USA) for 15 min on ice. Subsequently, the cell suspension was incubated with magnetic nanoparticles at 6 - 12 °C for 30 min. The cell suspension was loaded into an Imagnet column (BD), and the unlabeled cells that passed through the column were collected (Lin^−^ fraction). The column was then washed twice with 1 mL of buffer, and the remaining Lin^−^ cells were collected.

### Cell staining

To identify stem/progenitor hematopoietic stem cell, Lin^−^ cells were stained with monoclonal antibodies conjugated to different fluorochromes. These antibodies included: Sca-1(BD), CD133 (eBioscience, San Diego, CA, USA), c-kit (BD), Thy-1^lo^ (BD) and the appropriate isotype controls. HSCs were defined as c-kit^+^, Thy-1^lo^, Lin^−^, Sca-1^+^ (KTLS) and CD133^+^ in this study. No differences were found in the fluorescent intensity among the groups (data not shown). The expression of the intracellular pluripotency markers was also analyzed by labeling cells with three fluorochrome-conjugated antibodies: Oct3/4, Sox-2 and Nanog (BD), according to the manufacturer’s instruction.

### Cell cycle analysis

Cell cycle distribution was evaluated by flow cytometry. Prior to staining, 1×10^6^ cells were washed with phosphate buffered saline (PBS). The cells were treated with 50 μL of RNase (1 mg/mL) and 100 μL propidium iodide (PI, 400 μg/mL) (Sigma-Aldrich) for 30 min at 37 °C in the dark. The fluorescence of the stained cells was analyzed by flow cytometry, and the relative percentage of gated cells in each cell cycle phase was determined [23].

### Measurement of cytokine levels

Cytokine (IL-12p70, TNF, IFN-y, IL-10 and IL-6) levels were measured systemically and locally by flow cytometry using a Cytometric Bead Array – Mouse Inflammation Kit, according to the manufacturer’s instructions (BD). For these analyses, a typical forward and side scatter gate was set to exclude aggregates; a total of 5000 events in the gate were analyzed using FACSCanto II and FACSDiva Software (BD). The samples were quantified by comparison with standard curves of recombinant mouse cytokines using FCAP Array software (BD). The results were expressed as pg/mL.

### Measurement of intracellular ROS and hROS

ROS analysis was performed by flow cytometry as previously described [15]. Dihydroethidium (DHE, 160 μM) and 2’,7’-dichlorofluorescein diacetate (DCF, 20 mM) were added to the cell suspension (10^6^ cells) and incubated at 37 °C for 30 min in the dark to estimate the intracellular superoxide (•O_2_^−^) or hydrogen peroxide (H_2_O_2_) concentration, respectively. The measurement of nitric oxide (NO) was performed as previously described [24]. Briefly, the NO-sensitive fluorescent probe 4,5-diaminofluorescein-2/diacetate (DAF, 2 μM) was added to cell suspension (10^6^ cells) and incubated at 37 °C for 180 min in the dark. Highly reactive oxygen species (hROS), such as hydroxyl radical and peroxynitrite, were selectively detected by 2-[6-(4′-hydroxy) phenoxy-3H-xanthen-3-on-9-yl] benzoic acid (HPF). The cells were then washed, resuspended in PBS and analyzed by flow cytometry (FACSCanto II). The data were acquired using the FACSDiva software (BD) and overlay histograms were analyzed using FCS Express software trial (De Novo). For the quantification of DHE, DCF, DAF and HPF fluorescence, the samples were acquired in duplicate and 10,000 events were used for each measurement. The cells were excited at 488 nm; DHE fluorescence was detected using a 585/42 bandpass filter, and DCF/DAF/HPF fluorescence was detected using a 530/30 bandpass filter. The data are expressed as the median fluorescence intensity.

### Identification of oxidative stress sources

The cells were incubated in the presence of the inhibitors based on previous experiments [25] and ROS production was analyzed using DHE and DCF probes following established protocols from our laboratory [23]. The inhibitor concentrations were as follows: 100 μM clotrimazole for 10 min, 600 μM apocynin for 10 min, 100 μM allopurinol for 10 min, and 1 μM Carbonyl cyanide 3-chlorophenylhydrazone (CCCP) for 45 min. To investigate a decline in the capacity of the antioxidant enzyme system, 3 inhibitors were used: superoxide dismutase (SOD) through 500 μM sodium diethyldithiocarbamate (DDC) for 16 h, CAT through 20 mM 3-Amino-1,2,4-triazole (3-AT) for 2 h and glutathione peroxidase (GPx) through 50 μM mercaptosuccicinic acid for 2 h.

### Measurement of oxidized DNA by alkaline comet assay

DNA damage was assessed using alkaline single cell gel electrophoresis (the alkaline comet assay), following established protocols from our laboratory [23]. In brief, histological slides were pre-coated with 1.5 % normal melting point agarose in PBS in a water-bath at 65 °C. Subsequently, 20 μL of the cell suspension was embedded in 100 μL of 0.5 % low melting point agarose in PBS at 37 °C and spread on agarose-precoated slides using coverslips. Then, the slides were placed in an electrophoresis chamber filled with freshly prepared alkaline buffer (300 mM NaOH, 1 mM EDTA, pH > 13) for 40 min at 4 °C, and electrophoresed at 300 mA and 20 V for 30 min. Subsequently, the slides were neutralized with a 0.4 M Tris buffer (pH 7.5) for 5 min, washed with cold distilled water and allowed to dry at room temperature for 1 h. Migration of the DNA fragments towards the anode creates a comet ‘tail’ visualized by staining with ethidium bromide (20 μg/mL, Sigma-Aldrich). Immediately afterwards, images were obtained at a magnification of 200x using a fluorescence optical microscope (Nikon Eclipse Ti, Melville, NY, USA) equipped with excitation (510–550 nm) and barrier (590 nm) filters. The coded images were acquired using a CCD camera (Nikon) and analyzed using the CASP program (public domain) to determine the % DNA in tail and the tail moment parameters.

### Determination of cell viability, apoptosis and necrosis

Apoptotic HSCs were quantified by annexin V-FITC and propidium iodide (PI) double staining using the annexin V-FITC apoptosis detection kit (BD). In brief, the cells were washed twice with PBS and adjusted to 500 μL in binding buffer (5×10^5^ cells). Then, annexin V–FITC and PI were added to cell suspension, and the cells were gently vortexed. The cells were then incubated for 15 min at room temperature (25 °C) in the dark. Finally, the cells were analyzed by flow cytometry using a FACSCanto II (BD). Necrotic cells were defined as annexin V^−^/PI^+^, late apoptotic or secondary apoptotic were defined as annexin V^+^/PI^+^, and annexin V^+^/PI^−^ cells were recognized as early or primary apoptotic cells [23].

### Measurement of telomere length

Telomere length was measured with the telomere PNA kit/FITC (DAKO Denmark, Glostrup, DK). In brief, the sample DNA was denatured for 10 min at 82 °C either in the presence of hybridization solution without probe or in hybridization solution containing a fluorescein-conjugated PNA telomere probe. The hybridization then took place in the dark at room temperature overnight, followed by two 10-min post-hybridization washes with a wash solution at 40 °C. The sample was then resuspended in the appropriate buffer for further analysis by flow cytometry. The DNA Staining Solution included in the kit was used for the identification of G0/1 cells. After flow cytometry analysis, the data obtained were used to determine the relative telomere length (RTL). The RTL value was calculated as the ratio between the telomere signal of each sample and the control cells (1301 cell line, European Collection of Cell Cultures) with correction for the DNA index of G0/1 according to manufacturer’s instructions.

### Assessment of lysosomal content

Quantification of the cellular lysosomal mass was carried out by flow cytometry. The cells were stained with acridine orange (AO) as described before [26]. Briefly, 0.2 mL of cell suspensions were gently admixed with a solution containing 1 mg/ml of AO (Invitrogen, Eugene, OR), 1 mM EDTA-Na, and 0.15 M NaCl in phosphate-citric acid buffer (pH 7.4) was added for 10 min. The photomultiplier settings on the flow cytometer were adjusted to detect the green fluorescence signal of AO (mostly due to nucleic acid staining) on the 530/30 nm bandpass filter and the orange fluorescence signal (due to lysosomal staining) on the 585/42 nm bandpass filter.

### Senescence associated β-galactosidase (SA-β-gal) activity analysis

HSC senescence activity was determined using a SA-β-gal staining kit (Sigma-Aldrich) according to the manufacturer’s instruction. Briefly, the cells were washed with PBS and fixed in 2 % formaldehyde–0.2 % glutaraldehyde. Then, the cells were washed and incubated at 37 °C overnight with fresh SA-β-gal staining solution (1 mg 5-bromo-4-chloro-3-indolyl- β -D-galactopyranoside [XGal] per mL, 40 mM citric acid–sodium phosphate [pH 6.0], 150 mM NaCl, 2 mM MgCl_2_, 5 mM potassium ferrocyanide, 5 mM potassium ferricyanide). Senescent cells were identified as blue-stained cells by standard light microscopy, and a total of 1000 cells were counted in random fields on a slide to determine the percentage of SA-β-gal–positive cells. Photography of cells was performed on microscope (Nikon Eclipse Ti, Melville, NY, EUA) equipped with CCD camera (Nikon).

### Statistical analysis

Data are presented as mean ± SEM. The normality of the variables was evaluated using the Kolmogorov–Smirnov test. Analyses of variance (ANOVA) were performed using Graphpad Prism 6 software followed by Tukey’s multiple comparison tests to establish statistical significance between experimental groups at the *p* <0 .05 (*) level.

## Results

### Aging stimulates cell cycling and myeloid skewing

To evaluate the impact of aging on HSCs (KTLS/CD133^+^), we determined the number of cells and proliferation by cell cycle analysis (Table 1) and complete blood count (CBC) (Table 2). We observed a 3.3-fold increase in the number of HSCs during the lifespan (*p* < 0.05) when comparing aged and young mice. The cell cycle profile revealed an increased proliferation rate in HSCs from aged mice compared with young and middle mice, indicating that more HSCs began cycling with age (Table 1). Moreover, we observed a change in the number of mature hematopoietic cells in the peripheral blood. The increased engraftment of the myeloid lineage (1.6-fold, *p* < 0.05) and loss of lymphopoiesis support (−1.3-fold, *p* < 0.05) by aged HSCs was observed compared with young mice (Table 2). These data suggest that the natural aging process causes increased proliferation rates of HSCs, lymphoid senescence and myeloid skewing.

**Table 1:** Effects of aging on body weight, hematopoietic stem cell number, cell cycle profile and cytokines levels in C57BL/6 J mice

Parameters	Groups		
	Young	Middle	Aged
Body weight (g)	28 ± 1	29 ± 1	34 ± 1*
Hematopoietic stem cells (x10^6^)	1.7 ± 0.2	4.3 ± 1	5.6 ± 1^#^
Cell cycle (%)			
Sub G0	1 ± 0.2	4 ± 0.2	5 ± 0.5*
G0/G1	81 ± 2.6	75 ± 2.3	66 ± 2.9*
S/G2/M	18 ± 2.5	21 ± 2.6	29 ± 2.8*
Myeloid Cytokines (pg/mL)			
IL-6	23 ± 2	28 ± 2	63 ± 8*
TNF	111 ± 7	122 ± 7	227 ± 27*
IL-12p70	141 ± 11	142 ± 10	203 ± 14*
Lymphoid Cytokines (pg/mL)			
IFN-γ	3.2 ± 0.5	3.3 ± 0.6	4.1 ± 0.3
IL-10	181 ± 17	164 ± 16	149 ± 8

Values are means ± SEM for six to 11 animals per group. *p < 0.05 vs young and middle age; # *p* < 0.05 vs young (one-way ANOVA)

**Table 2:** The effect of aging on hematological parameters and systemic cytokines profile

Parameters	Groups		
	Young	Middle	Aged
Cell Blood Count (CBC)			
Red blood cell number (10^6^/ mm^3^)	4.4 ± 1	7.3 ± 0.2^#^	7.0 ± 0.3^#^
White blood cell number (10^3^/ mm^3^)	2.9 ± 0.4	2.8 ± 0.3	5.3 ± 1
Myeloid (%)	24 ± 4.3	27 ± 4.1	40 ± 4.2*
Lymphoid (%)	76 ± 4.3	73 ± 4.1	60 ± 4.2*
Myeloid Cytokines (pg/mL)			
IL-6	256 ± 30	348 ± 34	543 ± 37*
TNF	265 ± 19	398 ± 17	588 ± 80*
IL-12	153 ± 12	187 ± 8	172 ± 16
Lymphoid Cytokines (pg/mL)			
IFN-γ	69 ± 1.5	77 ± 2.3	75 ± 3.3
IL-10	516 ± 34	586 ± 22	573 ± 19

Values are means ± SEM for six to 11 animals per group. *p < 0.05 vs young and middle age; #*p* < 0.05 vs young (one-way ANOVA)

### Inflammatory cytokines are increased during aging

Aging is characterized by an increase in the levels of pro-inflammatory markers, which may contribute to impaired bone marrow HSC function and result in a state of chronic inflammation or “inflamm-aging” [27]. The pro-inflammatory cytokines IL-12p70, IL-6 and TNF can act as growth factors to stimulate proliferation. The data obtained by flow cytometry showed both locally (Table 1) and systemically (Table 2) increased levels of myeloid pro-inflammatory molecules in aged mice compared with young mice. However, no differences were found in the concentrations of IL-10 and IFN-γ, which could be expected to counteract the effects of pro-inflammatory cytokines.

### ROS production are augmented during aging

**Fig. 1:**
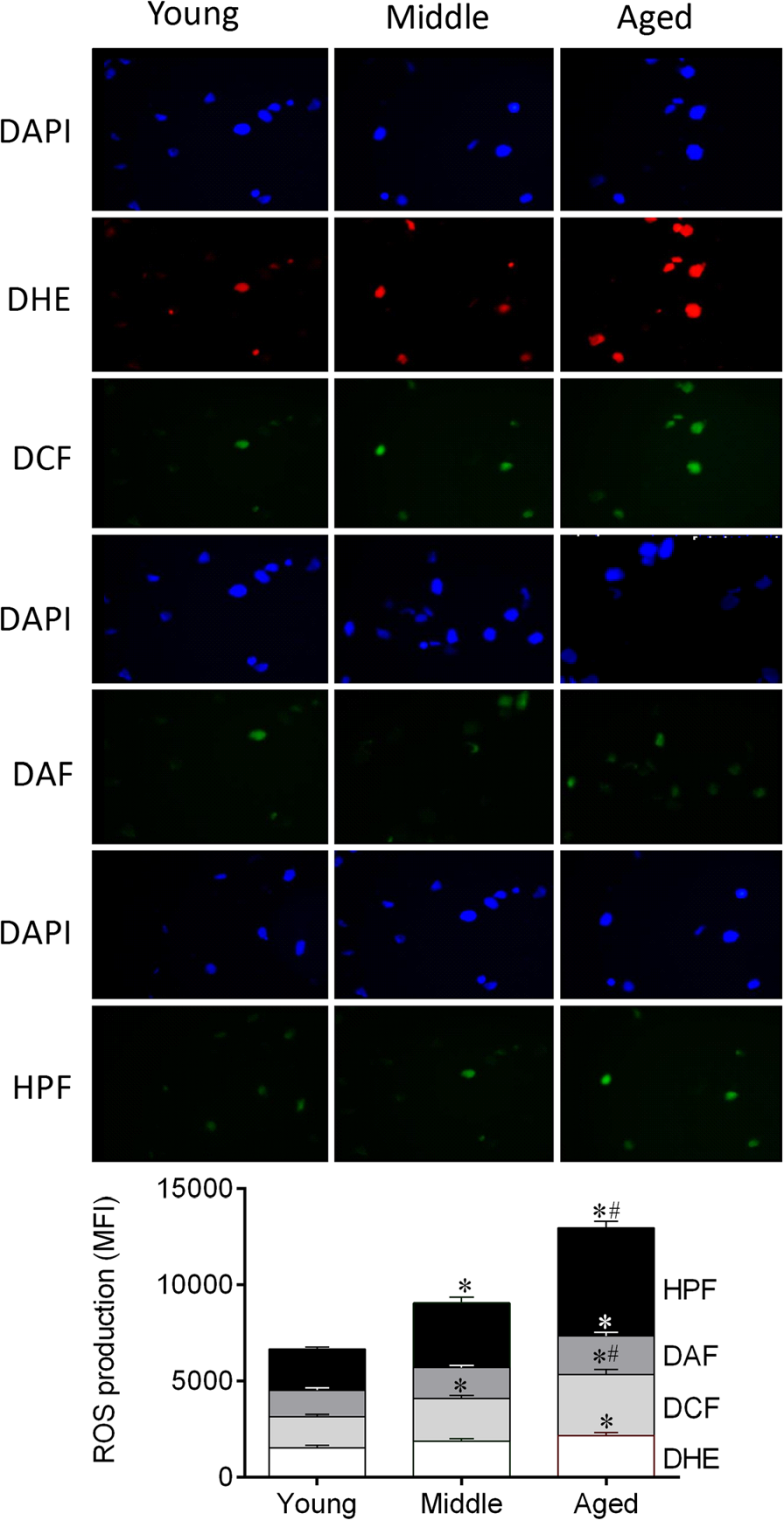
ROS generation and oxidative stress pathways are involved in aging. A- ROS production was assessed by DHE, DCF, DAF and HPF staining. Top panel shows representative images of HSCs; the aged group presented change in the number of cells that stained ROS-positive compared with young animals. Bar graph shows the difference on MFI of the ROS measured by flow cytometry among the groups. Values are means ± SEM, *p < 0.05 vs. young. ^#^ *p* < 0.05 vs. middle

Based on previous experiments showing that high levels of ROS represent a key mechanism for intrinsic HSC dysfunction [14, 28], we evaluated the intracellular ROS levels in HSCs in the three groups of animals. As illustrated in the top panel and summarized in the bar graphs of Fig. 1, we observed a clear age-related increase in ROS production. Specifically, our data showed significant increases in the levels of •O_2_^−^ and NO only in aged HSCs (1.4- and 1.6-fold, respectively) and augmentation in the H_2_O_2_ and in the hROS levels in both middle (1.2-, 1.4-fold, respectively) and aged (2-, 2.6-fold, respectively) compared with young HSCs (*p* < 0.05). This imbalance between ROS production and degradation could lead to genomic instability and, consequently, permanent changes in the genetic material.

### Sources of ROS and antioxidant enzyme capacity in HSCs

**Fig. 2:**
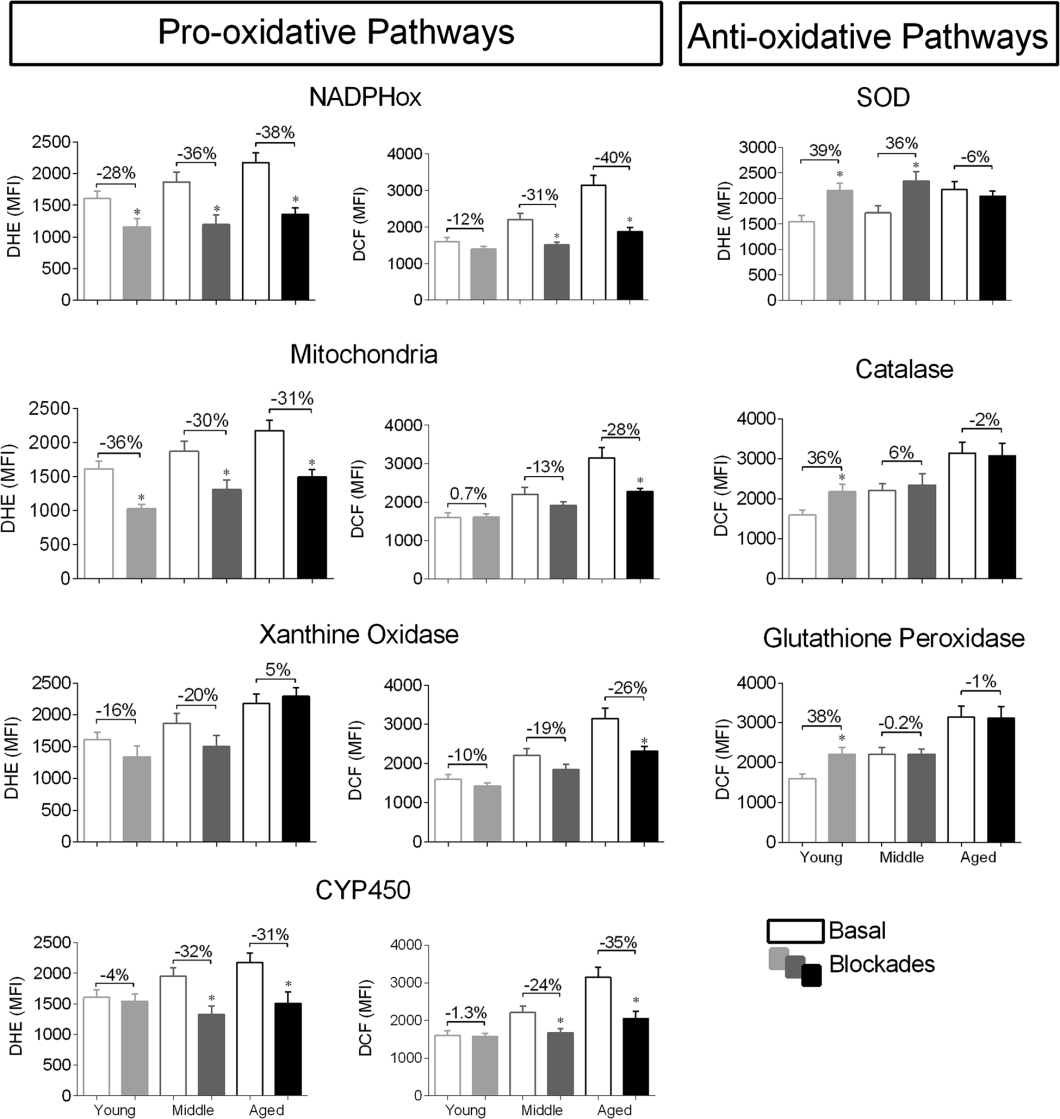
Identification of the main sources of ROS production and antioxidant enzyme capacity by oxidative stress pathway analysis. Bar graphs show the effect of different oxidant proteins inhibitors on ROS production and antioxidant proteins inhibitors on ROS reduction. ROS levels were analyzed by DHE and DCF fluorescence intensity. Unshaded bars represent basal ROS levels, and shaded bars show ROS levels after blockade. Abbreviations: NADPH oxidase (NADPHox), xanthine oxidase (XO), cytochrome P450 (CYP450), superoxide dismutase (SOD), catalase (CAT), gluthatione peroxidase (GPx). Values are means ± SEM, *p < 0.05 vs. matched basal

Considering that little is known about which pathways are involved in ROS production by HSCs and the stimuli of cell intrinsic alterations that trigger HSC aging, we evaluated the relative contributions of different pathways, which could lead to augmented ROS production and/or the loss of antioxidant capacity. To achieve our goal, we blocked the main sources of ROS and measured the fluorescent intensities of DHE and DCF. Figure 2 shows the average changes in pro-oxidative and anti-oxidative pathways in the three different age groups. We observed that in young animals the mitochondria and NADPHox represented the major pro-oxidative sources in HSCs. In the middle group, CYP450 contributed significantly in addition to these two sources. In aged HSCs, all of the investigated sources in this study actively contributed to the high levels of ROS production (NADPHox, mitochondria, CYP450 and XO). In a separate set of experiments, to investigate the involvement of the anti-oxidative pathways in the process of aging, we blocked three important enzymes: SOD, CAT and GPx. Our data showed high activity of these three anti-oxidative pathways in HSCs from young animals. In contrast, HSCs from middle mice exhibited a significant impairment in CAT and GPx function, which was more greatly impaired in cells from aged mice that showed reduced activity in the entire system compared with HSCs from young mice.

### DNA damage and apoptosis increases with aging

**Fig. 3:**
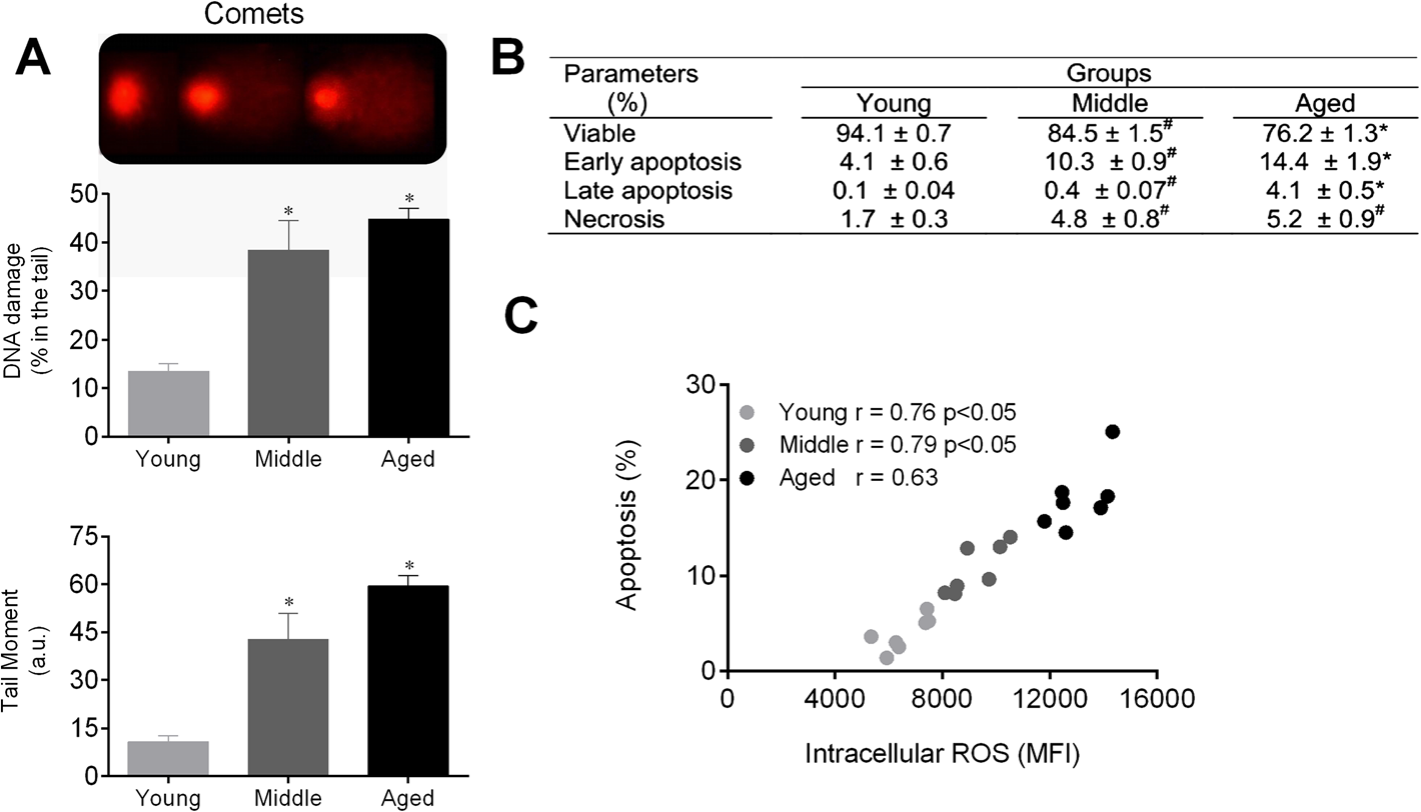
Augmented ROS leads to DNA damage and apoptosis of HSCs during aging. **a** The top panel shows typical photographs of comets with higher DNA fragmentation in the middle and aged groups. Bar graphs show the percentage of DNA in the tail and the tail moment. **b** Table shows the average percentage of viable cells, early apoptosis, late apoptosis and necrosis detected by annexin-V and PI staining. **c** Correlation between intracellular ROS levels and apoptosis. Values are means ± SEM, *p < 0.05 vs. young. ^#^ *p* < 0.05 vs. middle

In this study, we used the alkaline comet assay to evaluate DNA damage, expressed by the percentage of DNA in the tail which represents the number of fragments that migrated during electrophoresis, and the comet tail moment, which is an index of both the migration of the genetic material and the relative amount of DNA in the tail [15, 23, 24]. Figure 3a shows a remarkable increase in DNA fragmentation in HSCs from middle (4.2-fold) and aged (5.9-fold) mice compared with young mice in the tail moment parameter (*p* < 0.05). Moreover, HSCs from middle and aged mice showed more DNA in the tail (2.8- and 3.3-fold, respectively) compared with HSCs from young mice (*p* < 0.05). High ROS levels are frequently associated with an increase in DNA fragmentation and, consequently, more apoptosis [15, 23, 24].

An analysis of apoptosis can be used to assess aging and survival in the hematopoietic compartment. Figure 3b shows that cell viability was reduced throughout the life span. Middle and aged mice showed a significant increase in the percentage of cells in early apoptosis (10 and 14 %, respectively), defined by annexin-V^+^/PI^−^, compared with young mice (4 %). In contrast, only aged HSCs demonstrated a remarkable change in late apoptosis (4 %), defined by annexin-V^+^/PI^+^, compared with young (0.1 %, *p* < 0.05) and middle (0.3 %, *p* < 0.05) mice. Necrosis, defined as annexin-V^−^/PI^+^, increased in middle and aged HSCs (~5 %) compared with the young group (~1.5 %, *p* < 0.05). The increase in ROS levels during the life span was positively correlated with the increase in apoptosis (Fig. 3c). These data suggest that increased ROS production in aged HSC may be involved in the apoptotic signaling pathway.

### Cellular senescence increases during the aging process

**Fig. 4:**
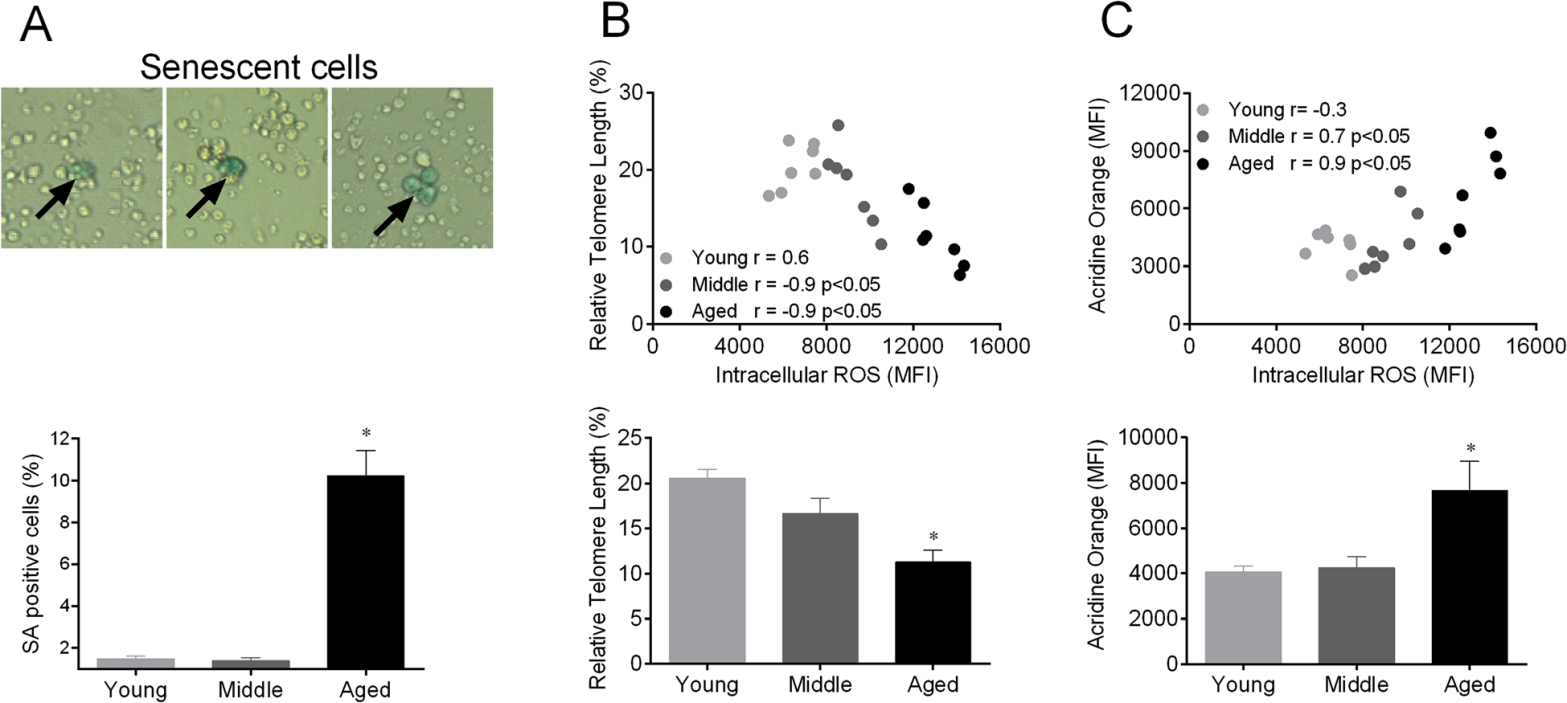
Aging leads to molecular and cellular senescence of HSCs. **a** The top panel shows typical senescent cells with blue cytoplasm (arrow) and the bar graph shows the percentage of positive cells quantified by cell counting (bottom panel). **b** Top panel shows the correlation between intracellular ROS levels and acridine orange fluorescence. The bar graph shows the median fluorescence intensity of acridine orange (bottom panel). **c** The top panel shows the correlation between the intracellular ROS levels and relative telomere length (RTL). Bottom panel show the percentage of RTL. Values are means ± SEM, *p < 0.05 vs. young

To investigate the effects of aging on HSCs, we performed the SA-β-gal, which is one of the most widely used biomarkers for aging cells [15]. As illustrated in Fig. 4a (, top panel), the cytoplasm of senescent cells stains blue allowing easy identification. The aging process induced a remarkable increase in the percentage of SA-β-gal positive HSCs (~10 %) compared with young and middle (~1.5 %) animals, as summarized in the bar graph (Fig. 4a). To investigate senescent characteristics, we evaluated telomere length and lysosomal mass. Telomere shortening limits the cell proliferation capacity to a finite number of cell divisions. The accumulation of DNA damage and telomere dysfunction can contribute to the decline of stem cell maintenance and function [8]. In the present study, the HSC telomere length was shortened in approximately 80 % (*p* < 0.05) of aged and 23 % of middle mice compared with young mice, suggesting reduced telomerase activity in aged mice (Fig. 4b). We also observed a remarkable inverse correlation between the intracellular ROS levels and the RTL in HSC from middle and aged (*r* = −0.9, *p* < 0.05) mice (Fig. 4b, top panel). Then, we investigated whether the senescence could also result from changes in lysosomal mass. Our data indicated an increased side scatter (SSC) profile in the HSC aged group, which is an indicator of cellular granularity (data not shown). Finally, we also performed a flow cytometry analysis of AO fluorescence, and the aged group showed a dramatic increase of ~90 % compared to middle and young mice (Fig. 4c). Therefore, the increase in both SSC and AO fluorescence is indicative of augmented lysosomal content in aging cells [26]. Moreover, the increase in AO fluorescence correlated closely with the increase in ROS levels observed in the aged group (Fig. 4c, top panel). Altogether, these data indicate that high ROS levels can trigger senescence.

### Cell fate and self-renew are impaired in HSC bone marrow niche

**Fig. 5:**
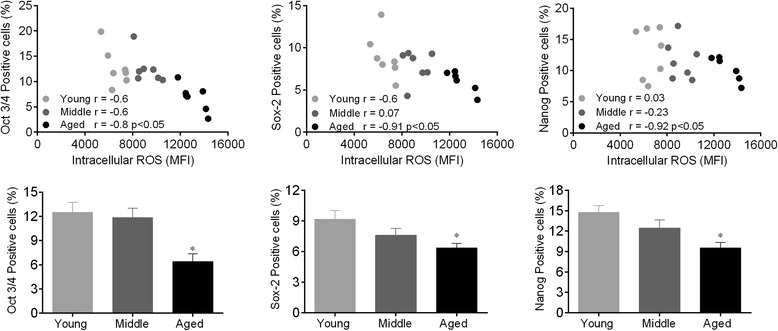
Pluripotency markers are downregulated by augmented ROS production during aging. The top panels show the correlation between intracellular ROS and ONS transcription factors. Bar graphs show the percentage of positive ONS cells. Values are the means ± SEM, *p < 0.05 vs. young. Abbreviation: Oct-3,4/Nanog/Sox-2 transcription factors (ONS)

ROS-induced DNA damage is associated with genomic instability and, consequently, the loss of regenerative capacity, self-renewal dysfunction and cell cycle arrest of HSCs. We tested the hypothesis that the high ROS levels observed in aged HSCs may influence the expression profile of the Oct-3/4, Sox-2 and Nanog transcription factors (ONS) derived from young, middle and old bone marrow. As illustrated in Fig. 5 (bottom panels), our data showed that ONS expression was significantly decreased in aged HSCs compared with the middle and young groups (Oct-3/4: −50 %; Sox-2: −30 %; Nanog: −23 %, *p* < 0.05). This finding in aged HSC supports the idea that the loss of the regenerative capacity and self-renewal of these cells is correlated with the high ROS levels (Fig. 5, top panels).

## Discussion

In the present study, we investigated age-related changes in HSC function and found an increase in the number of cells, prevalence of cells in the cycling phases, and a myeloid skewing of committed hematopoietic cells that was induced by the high levels of pro-inflammatory cytokines. The novel finding of this study was the characterization of the major sources of ROS production in HSCs, which occurred in an aging-dependent manner. Our data show that the consequences of ROS accumulation in HSCs include genotoxic damage, apoptosis, senescence and the loss of multipotency of these aged cells.

### HSC number, cell cycle, cbc and cytokines

Previous experimental and clinical data have shown that aging promotes the increase of HSCs number in the bone marrow [29, 30]. In our study, we also observed high HSC proliferative activity that was associated with aging, which in turn was associated with an approximately 10 % increase in the number of cells in the S/G2/M phases of cell cycle in aged mice. Initially, we speculated that this augmented proliferative activity might be a fundamental compensatory mechanism to maintain the lymphopoiesis and self-renewal functions of these HSCs. However, we detected that the aging process resulted in a decrease of lymphopoiesis and in myeloid skewing. Moreover, our data revealed a change in CBC and an increase in the myeloid-derived cytokine levels (IL-6, TNF, IL-12), without alterations in the lymphoid-derived cytokines (IFN-γ, IL-10). Our data are in accordance with other studies, which showed that aged HSC are more actively cycling and preferentially differentiating toward myelopoiesis [7, 8]. Because oxidative stress is involved in the induction of systemic and local myeloid cytokines production [31] and because HSCs are extremely sensitive to the extensive accumulation of ROS [32], we used flow cytometry to investigate whether excessive ROS production could contribute to the amplification of myeloid skewing over time.

### ROS overproduction in aged HSCs

It is known that bone marrow HSCs with low ROS activity exhibit normal lymphoid production, which allows them to maintain their quiescence and self-renewal potential [33]. Some mechanisms appear to protect HSC from oxidative stress, such as antioxidant enzyme status and preferential anaerobic glycolysis instead of oxidative phosphorylation to ATP production [34]. In our study, we observed that the protection against this oxidative stress was lost during the aging process; we detected higher levels of ROS production in aged HSCs with different probes. Aged mice exhibited high levels of all molecules, as indirectly measured by flow cytometry: •O_2_^−^, H_2_O_2,_ NO, including the strongest oxidant produced in biological systems: •ONOO^−^ and •OH^−^. These data complement and strengthen those obtained in previous studies regarding ROS detection [28, 35] and highlight the possible contribution of oxidative stress for myeloid skewing over time.

### Sources of ROS and antioxidant enzyme activity

Several studies have demonstrated that all cell types produce ROS in multicellular organisms [36, 37]; although the mitochondria has traditionally been considered the main source of intracellular ROS [38], other enzymatic systems also contribute to ROS generation, including NADPHox [39], XO, cyclooxygenases, CYP450 complex, nitric oxide synthase and lipoxygenases [40]. However, prior to the present study, little was known about which sources of ROS are involved in aging and, more specifically, which pathways are involved in bone marrow HSCs. For this purpose, we tested the hypothesis that at least four main sources could be involved in ROS overproduction during the life span. The data obtained through the use of specific pharmacological blockers confirmed the idea that the mitochondria and NADPHox are the major sources of ROS in young HSCs, as reviewed by Urao and Ushio-Fukai [16]. However, in the present study, we expanded the time-course, and our data revealed a different profile for ROS sources. For instance, CYP450 played a relevant role in both middle and aged HSCs, and XO was an important source in aged HSCs.

The process of aging generates an “inflamm-aging” environment, in which higher levels of ROS may activate inflammatory mediators and vice-versa [41]. We observed different complexes contributing to an increase in oxidative stress and the augmentation of local and systemic pro-inflammatory cytokines. Our data are consistent with those of other studies demonstrating that elevated ROS activity in aged bone marrow has the ability to induce local mediators of inflammatory production [31] and that aging increases the systemic cytokine levels [33].

In relation to antioxidant enzymes, we observed that in HSCs from middle mice, the SOD activity was preserved but the CAT and GPx were impaired, which could explain the accumulation of H_2_O_2_ and the maintenance of •O_2_^−^ levels compared with young HSCs. Conversely, in aged HSCs, there was an impairment of all three antioxidant enzymes, which may directly reflect ROS augmentation. As a consequence of this process, high intracellular ROS production may be involved in cellular signaling and may regulate different mediators [42]. Among them, the FoxO transcription factors appear to be critical mediators of the cellular responses to oxidative stress, since the lack of FoxO subtypes in HSCs down-regulates the expression of antioxidant enzymes (such as SOD and CAT) and consequently leads to an increase in ROS levels within the HSC compartment [43]. Based on these data, we speculated that aging may impair the expression of the FoxO family and consequently diminish the anti-oxidative enzyme levels.

### DNA damage and apoptosis

ROS are highly reactive and can oxidize nucleic acids, proteins and lipids [23, 24]. We performed an alkaline comet assay to assess single- and double-strand breaks in DNA from young, middle and aged HSCs. Our data showed a strong correlation among ROS production, DNA damage and apoptosis. As expected, DNA fragmentation, cell death and ROS production were aggravated in the aged HSCs. Our data are in agreement with those of Rossi et al. [8], who investigated the HSC reserves and function in aged mice, which are deficient in several genomic maintenance pathways, including nucleotide excision repair, telomere maintenance and non-homologous end-joining with elevated levels of apoptosis. Moreover, Jang et al. [14] demonstrated that although low levels of ROS are involved in the maintenance of quiescence in HSCs, higher levels of ROS contribute to greater proliferation, senescence or apoptosis, which in turn lead to the premature exhaustion of self-renewal in these cells. We observed that high ROS production leads to the accumulation of DNA damage. Cells in which the damage can be repaired may continue through the cell-cycle, whereas those that have suffered irreparable genotoxic oxidative damage undergo apoptosis or cell-cycle arrest [44]. Therefore, the causative role of augmented ROS production in the loss of cell viability is substantiated by increased DNA fragmentation. This is favored by an augmented cell proliferation rate, suggesting that ROS can act as secondary messengers to modulate multiple cell signaling pathways.

### HSC senescence

Oxidative stress is a determining factor of cellular senescence, a biologically active response that contributes to tissue aging through at least two mechanisms [8, 45]: first, intrinsically, through the inability to further proliferate to replace tissues with new cells, and second, by up-regulating genes that encode inflammatory cytokines, growth factors and extracellular-matrix-degrading enzymes [45]. Then, we investigated the impact of aging on cellular senescence using a SA-β-gal assay and AO fluorescence, which indirectly reflects the lysosome malfunction; when the property of autophagy is lost, the lysozymes accumulate in the aging cells [26, 46]. In the present study, we found that both the number of SA-β-gal positive HSCs and AO fluorescence were increased in aged mice. These results indicate that cell senescence increases the level of β-galactosidase activity and lysosomal content in aged HSCs.

To confirm senescence using a second, well-established procedure, we investigated the shortening of telomeres. Indeed, dysfunctional telomeres have been found, in vivo, in senescent cells, and the loss of telomerase function causes the senescence and physiological impairment of many tissues [45]. In our study, aged HSCs showed shorter telomeres than HSCs from the young group. However, it should be noted that not all studies have detected the association between telomere shortening and senescence [35] because cellular senescence may occur in an independent manner [47]. Furthermore, a decline in autophagic capacity caused by aging leads to an accumulation of damaged mitochondria and, consequently, to an increase in ROS production, which can stimulate the secretion of pro-inflammatory cytokines and accelerate the aging process, the so-called “inflamm-aging” condition [41]. Additionally, in attempt to answer a possible question whether ROS contributes directly to DNA damage and cellular senescence, we designed a new in vitro protocol focused in this issue. Briefly, we verified that a sub-lethal dose of H_2_O_2_ (at 100 μmol/L for 12 h) augmented the rate of sub-G0 cells and SA-β-gal–positive cells (2-fold in relation to H_2_O_2_ at 10 μmol/L) which was rescued by ascorbic acid pretreatment (50 mg/mL) for 30 min (data not shown).

### HSC ONS transcription factors

The control of cellular fating is a complex process in which the cell must coordinate many different signals [48]. Considering the importance of ROS in cellular signaling and the regulation of gene expression, it is feasible that ROS are involved in cell differentiation [42]. We next evaluated the age-related changes in cell plasticity and self-renewal. The uniform expression of hematopoietic markers (KTLS/CD133^+^ fraction: c-kit^+^ Thy-1^lo^Lineage^−^Sca-1^+^CD133^+^) in each group, as observed in this study, indicates homogeneity in the isolated bone marrow-derived HSC population and does not necessarily correlate with plasticity. Conversely, the progressive loss of pluripotent transcription factors is indicative of the age-related loss of multipotency. For instance, it is well documented that the combinatorial transcription factor interaction network of ONS is associated with pluripotency and stemness in embryonic stem cells [35]. Interestingly, our results revealed age-related changes in the ONS expression profile. Similarly, Assumda et al. [49] found a decreased expression of ONS markers in bone marrow-derived mesenchymal stem cells from old rats. Moreover, a recent study showed stemness loss and lineage commitment of multipotent populations induced by the inflammatory cytokine secretion of senescent-like cells [50]. Our data suggest that increased levels of intracellular ROS diminish ONS positivity, as observed through the negative correlation between ONS and ROS (Fig. 5). In accordance with this hypothesis, recent studies have demonstrated the importance of ROS in cell differentiation in the mouse and human [51, 52] and that human embryonic stem cell pluripotency can be improved through reduced levels of ROS [53]. Although the exact role of ROS in stemness has yet to be clarified, it is now clear that redox homeostasis should be strictly regulated to avoid HSC exhaustion.

### Cell cycling and premature exhaustion

HSCs preferably remain quiescent in bone marrow, making them especially susceptible to accumulation of DNA damage, which contributes to the gradual decrease in their function with age [54]. Genotoxic oxidative stress causes loss of HSC self-renewal capability [55] since they quit quiescence and then differentiate, preventing the propagation of genetically unstable HSCs [56–59]. In response to genotoxic oxidative stress, HSCs can undergo extensive proliferation caused by increased levels of extrinsic [60] and intrinsic [61] factors that play important roles in determining HSCs fate. In addition, this process could lead to HSC exhaustion and myeloid skewed differentiation [14, 56, 62, 63].

The mechanisms involved in repair of DNA damage are initiated when aged HSC re-enter the cell cycle [54, 64, 65] and their fate could result in cellular dysfunction leading to the homeostatic failure of bone marrow HSC pool [66–68]. It must be taken into account that augmented ROS production can inhibit HSC self-renewal not only causing differentiation but also leading the HSC to senescence [68–70] and/or apoptosis [67], as it was demonstrated in the present study. Attempting to answer whether ROS compromises HSC self-renewal, we repeated the previous in vitro protocol but now focusing in ONS expression and founded that ascorbic acid pretreatment (50 mg/mL) rescued ONS expression in HSC (data not shown).

## Conclusion

**Fig. 6:**
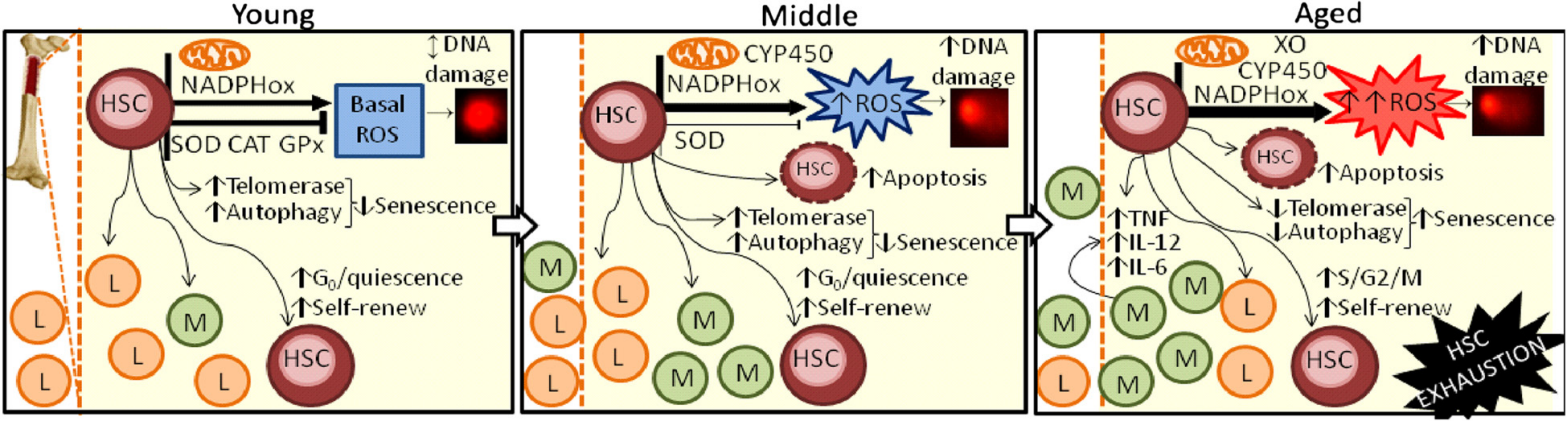
Scheme showing multiple age-related factors regulating hematopoietic stem cell function. Alterations in HSC function observed in aging: generation and main types of damage induced by ROS, different mechanisms of oxidative cellular/molecular damage and the potential role of antioxidants on HSC fate during the aging process. Lines with arrowhead indicate stimulation and lines with crosshead indicate inhibition. A line with a double arrowhead indicates a normal condition. Abbreviations: hematopoietic stem cells (HSC), reactive oxygen species (ROS), NADPH oxidase (NADPHox), cytochrome P450 (CYP450), xanthine oxidase (XO), lymphoid cells (L), myeloid cells (M), tumoral necrosis factor (TNF), interleukin-12p70 (IL-12), interleukin-6 (IL-6)

The lifelong potential of HSCs has led to the hypothesis that these cells could preserve both stemness function and integrity. However, the present study provides new evidence that cellular and molecular changes occur in HSCs during aging. We investigated aspects of HSCs, including self-renew, survival, differentiation, proliferation and mobilization. We observed that aged mice exhibited an increase in HSC number, myeloid skewing, DNA damage, fewer quiescent cells and more cells in S/G2/M phase of the cell cycle. Apoptosis and senescence may have increased to control age-dependent alterations. The scheme proposed in Fig. 6 emphasizes oxidative stress, the impairment in antioxidant defenses and inflammation as the most important stimuli leading to age-related changes observed in the bone marrow niche. Therefore, our model contributes to a better understanding of age-related changes in stem cells and may be helpful in explaining the degenerative changes observed in organs during aging.

## Abbreviations

(CAT): Catalase
(CYP450): Cytochrome P450
(NF-κB): Factor nuclear kappa B
(FOXO3): Forkhead Box O3
(GPx): Glutathione peroxidase
(HSC): Hematopoietic stem cell
(IFN-γ): Interferon-γ
(IL-12): Interleukin-12p70
(IL-6): Interleukin-6
(IL-10): Interleukin-10
(L): Lymphoid cells
(M): Myeloid cells
(NADPHox): NADPH oxidase
(ONS): Oct-3,4/Nanog/Sox-2 transcription factors
(p66shc): Proto-oncogene Src homologous-collagen homologue
(ROS): Reactive oxygen species
(SIRT1): Sirtuin 1
(SOD): Superoxide dismutase
(TNF): Tumoral necrosis factor
(XO): Xanthine oxidase
